# Chloroplast Genome Evolution and Codon Usage In the Medicinal Plant *Pothos chinensis* (Araceae)

**DOI:** 10.3390/genes16091017

**Published:** 2025-08-28

**Authors:** Hua Chen, Jisi Zhang

**Affiliations:** Liaoning Key Laboratory of Development and Utilization for Natural Products Active Molecules, Anshan Normal University, Anshan 114000, China; chenhua@asnc.edu.cn

**Keywords:** *Pothos chinensis*, chloroplast genome, codon usage bias, phylogeny

## Abstract

Background/Objectives: *Pothos chinensis* is commonly used as traditional medicine in China and India. Codon usage analysis is a good way to understand plants’ evolution. However, there is no report about the codon usage bias of chloroplast genomes in *P. chinensis*. Methods: In this study, the chloroplast genome of the medicinal plant *P. chinensis* was newly obtained. Comparative analyses, DNA barcoding investigation, codon usage bias, and phylogenetic reconstruction were conducted to reveal the chloroplast genome characteristics of *P. chinensis*. Results: The length of the chloroplast genome of *P. chinensis* was 165,165 bp. A total of 134 genes were annotated, i.e., 90 protein-coding genes, 36 transfer RNA genes, and eight ribosomal RNA genes. Compared to its sister group *Anthurium andraeanum*, the length of the large single-copy region (LSC) had been expanded, while the small single-copy region (SSC) had been contracted. Within *P. chinensis* and *P. scandens* there were no obvious differences in the length of LSC, SSC, and two inverted repeat regions. Based on Pi values, seven hypervariable regions of whole plastomes were identified. The analysis of codons showed that an average frequency of the 50 candidate genes was 35.30%, and these genes preferred A/U-ending codons. The average effective number of codon (ENC) value was 45.49, which indicated weak codon usage bias. ENCs had a highly significant positive correlation with GC3. Fourteen optimal codons had been identified, 11 of which ended with A/U. The results of the neutrality plot, ENC-plot, and PR2-plot analysis indicated that natural selection might have a significant impact on codon usage patterns. Conclusions: Taken together, our study unraveled the codon usage patterns in *P. chinensis* and provided valuable genetic information for the genus *Pothos*.

## 1. Introduction

The angiosperm family Araceae contains eight subfamilies, 144 genera, and 3645 species [1], and distributes in tropical and temperate regions [2,3,4]. Among the eight subfamilies, Pothoideae is the second largest subfamily with approximately 1010 species [1], and divides into Tribe Potheae (including *Pothos* L., *Pedicellarum* M. Hotta, and *Pothoidium* Schott) and Tribe Anthurieae (only *Anthurium* Schott) [2,3,5]. The largest genus *Pothos* contains approximately 75 species, and distributes in tropical and subtropical regions of Asia, Australia, Madagascar, and Polynesia, with five species in China [6]. Importantly, *Pothos* holds high importance as a traditional medicine in China and India, involving significant financial transactions and thus exhibiting robust economic value [7,8,9]. *P. chinensis* is a traditional medicinal herb to treat rheumatic numbness, traumatic injuries, and fractures [7]. The results of the integration of network pharmacology, serum pharmacochemistry, and metabolomics to investigate the anti-inflammatory mechanism of *P. chinensis* demonstrated that the anti-inflammatory modulatory network of this species was related to five metabolites, three metabolic pathways, seven targets, and four active components [8]. The Indian people use an infusion of the leaves of *P. scandens* as a bath for curing convulsions and epilepsy [9]. Recently, phylogenetic analysis strongly supported the sister relationships of *Pothos* and *Anthurium* based on chloroplast genomes [10]. Further, a previous study compared the chloroplast genomes of *P. scandens* and *Anthurium huixtlense*, and the results showed *P. scandens* with a unique contraction and expansion of inverted repeats (IRs) [11]. Compared with *A. huixtlense*, the ratio of transition to transversion mutations of *P. scandens* was a little higher. So far, the chloroplast genomic composition of another herb *P. chinensis* is far from being known and fully understood. Therefore, it is necessary to obtain the chloroplast genomes of *P. chinensis* and reveal the chloroplast genome evolution within *Pothos*.

The chloroplast genomes have provided valuable opportunities to the development of the specific DNA barcodes within a certain genus [12,13,14]. Authors such as Zhou et al. (2024) resolved the major clades within *Gastrochilus* based on the whole chloroplast genomes and identified the mutation hotspots to develop specific DNA barcodes [15]. In addition, codon usage bias in chloroplast genome is a widespread phenomenon in plants [16]. Codon bias analysis can reveal natural selection, mutation pressures and evolutionary tendency in chloroplast genomes, which provide crucial insights for evolutionary biology [17]. Different plants experienced diverse environments, and they have had different codon usage patterns mainly for the influencing factors including natural selection, mutation pressure, gene function, and gene length [18,19,20,21]. For instance, the investigation of the codon usage patterns in the chloroplast genomes within five *Caragana* species showed that the codon usage bias had high similarity in these species, and natural selection was the primary factor on codon usage bias rather than mutation pressures [22]. Here, studying codon usage patterns in chloroplast genomes of *P. chinensis* would improve our understanding of gene expression status and their evolutionary dynamics.

In this study, we newly sequenced, assembled, and annotated the chloroplast genome of *P. chinensis*. Moreover, we conducted comparative analysis of this information with two previously released chloroplast genomes of *Pothos* species, and performed DNA barcoding research, phylogenetic reconstruction, and codon usage bias. The present work is aimed at (1) exploring the general features of *Pothos* chloroplast genomes at intraspecific and interspecific levels, and (2) revealing the codon usage patterns and its influencing factors in *P. chinensis*. The results are expected to identify the chloroplast evolutionary patterns of *P. chinensis* and provide valuable genetic information for the genus *Pothos*.

## 2. Materials and Methods

### 2.1. Sampling and Sequencing

The leaf samples were collected from Libo County, Guizhou Province, China (25°20′57″ N, 107°46′37″ E). The voucher specimen (accession no. ZJS_2023070) was deposited in the specimen room of Anshan Normal University (https://www.asnc.edu.cn/, accessed on 1 January 2024), Contact: Ji-Si Zhang, E-mail: zhangjisi@asnc.edu.cn). Total genomic DNA was extracted from silica gel-dried leaves using the modified CTAB method [23]. An illumina paired-end (PE) library was prepared and sequenced in the Nanjing Novogene Biotechnology Co., Ltd., Nanjing, China.

### 2.2. Chloroplast Genome Assembly and Annotation

Near 8 Gb of 150 bp paired-end raw reads were generated and used for chloroplast genome assembly. Trimmomatic 0.39 [24] was used to organize and trim overrepresented sequences for obtaining the clean reads. The clean reads were assembled by using GetOrganelle v1.5 [25]. The chloroplast genome of *P. chinensis* was annotated using GeSeq [26] and Geneious v9.1.4 (http://www.geneious.com/, accessed on 26 December 2024) with *P. scandens* (MN046891) and *P. chinensis* (PP754463) as references. The annotated complete chloroplast genome of *P. chinensis* was deposited in GenBank (the accession number PV938952).

### 2.3. Structure and Sequence Divergence Analyses

In order to detect the potential expansion and contraction at the boundaries of the inverted repeat regions (IRs), the genes across the boundary regions of LSC/IRb/SSC/IRa were visualized using IRscope v3.1 [27]. Additionally, the Mauve v1.1.3 [28] plugin in Geneious v9.1.4 was employed to conduct the collinearity analysis with default parameters, which was used to detect the gene arrangement.

### 2.4. Evolutionary Hotspots and Phylogenetic Analysis

The hypervariable regions of three *Pothos* individuals were detected on the whole chloroplast genomes. The matrix was aligned using MAFFT v7 [29] and manually adjusted in BioEdit v7.0 [30]. The Pi values were calculated in DnaSP v6.12.03 with sliding window analysis by setting the step size to 200 bp and window length to 800 bp [31].

According to the previous molecular phylogenetic studies [10,32], 50 species of Araceae were selected to reconstruct the phylogenetic tree (Table S1). The whole chloroplast genome matrix was aligned using MAFFT v7 [29] and trimmed using Gblock 0.91b (https://www.biologiaevolutiva.org/jcastresana/Gblocks.html, accessed on 5 January 2002) to remove poorly aligned positions. In the ML analysis, the GTRGAMMA model was specified and bootstrap values were calculated on 1000 bootstrap replicates by RAxML [33]. In the MP analysis, a heuristic search with 1000 random addition sequence repeats were conducted, employing TBR branch switching by PAUP 4b10 [34]. All characters were treated as equal and unordered.

### 2.5. Calculation of Parameters Related to Codon Usage Bias

All protein coding sequences (CDSs) were selected as follows: (1) with the start codon (ATG) and with the stop codons (TAA, TAG, or TGA), (2) no stop codons appeared prematurely within the sequences, and (3) a length greater than 300 bp. Finally, 50 protein-coding genes were chosen and analyzed for the codon usage bias in the *P. chinensis* chloroplast genome.

The software CodonW 1.4.2 was used to calculate the Relative Synonymous Codon Usage (RSCU) and Effective Number of Codons (ENC). RSCU represents the relative frequency of codon usage in encoding a particular amino acid. An RSCU value greater than 1 indicates high preference, an RSCU value equal to 1 indicates no preference, and an RSCU value less than 1 indicates weak preference [35,36]. The analysis data was organized and analyzed using IBM SPSS 29.0 software. The total GC content (GC_all) of each gene coding region, as well as the GC content at the 1st (GC1), 2nd (GC2), and 3rd (GC3) codon positions, were calculated using the CUSP online program (http://www.bioinformatics.nl/emboss-explorer/, accessed on 6 March 2025).

### 2.6. Identification of Optimal Codons

Based on the ENC values, the 5 highest (10%) and 5 lowest (10%) ENC values among the 50 candidate genes of *P. chinensis* were selected to form the high expression group (5 genes) and the low expression group (5 genes), respectively. The RSCU values for both the high and low expression groups were calculated, and the difference in RSCU (ΔRSCU) between the two groups was determined. Codons with ΔRSCU greater than 0.08 were considered high expression codons. The intersection of high expression codons and high frequency codons (RSCU > 1) was taken, and the common codons were identified as the optimal codons [37].

### 2.7. Neutrality Plot Analysis

Neutrality plot analysis is a method to measure the factors which influenced codon bias. A scatter plot is drawn with GC12 (the average of GC1 and GC2) on the *Y*-axis and GC3 values of each gene on the *X*-axis. If there is a high correlation, all the points are distributed along the diagonal, indicating that the base usage patterns at various positions are similar and that the codon usage pattern is mainly related to mutational factors. Conversely, randomly distributed points indicate differences in base usage, suggesting a strong conservation of GC content in the sample and that natural selection has a greater influence on the codon usage pattern [38,39].

### 2.8. ENC-Plot Analysis

ENC is an important indicator for analyzing the overall level of codon bias in a gene. Genes with high expression levels exhibit strong codon bias and have lower ENC values, while genes with low expression levels use a wider variety of rare codons and have higher ENC values. A two-dimensional scatter plot is drawn with the actual ENC values on the *Y*-axis and GC3 values on the *X*-axis, and a standard curve is plotted using the expected ENC values, where ENC = 2 + X + 29/[X^2^+ (1 − X)^2^], and X represents the GC3 value. The closer a gene point is to the standard curve, the stronger the influence of the mutation on codon usage bias; the farther a gene point is from the standard curve, the stronger the influence of natural selection on codon usage bias [40].

### 2.9. PR2-Bias Plot Analysis

PR2-bias plot analysis involves the screening of the base composition at the third position of codons for amino acids encoded by four codons, avoiding the mutational imbalance between A/T and C/G at the third codon position. It follows the rule that no selection or mutational bias occurs when the content of two complementary bases is equivalent [41]. A scatter plot is drawn with A3/(A3 + T3) on the *Y*-axis and G3/(G3 + C3) on the *X*-axis. The central point (where A = T and C = G) represents unbiased usage, and the position of each point relative to the center indicates the direction and extent of bias in the gene [42,43].

## 3. Results

### 3.1. Chloroplast Genome Characters of P. chinensis

**Figure 1:**
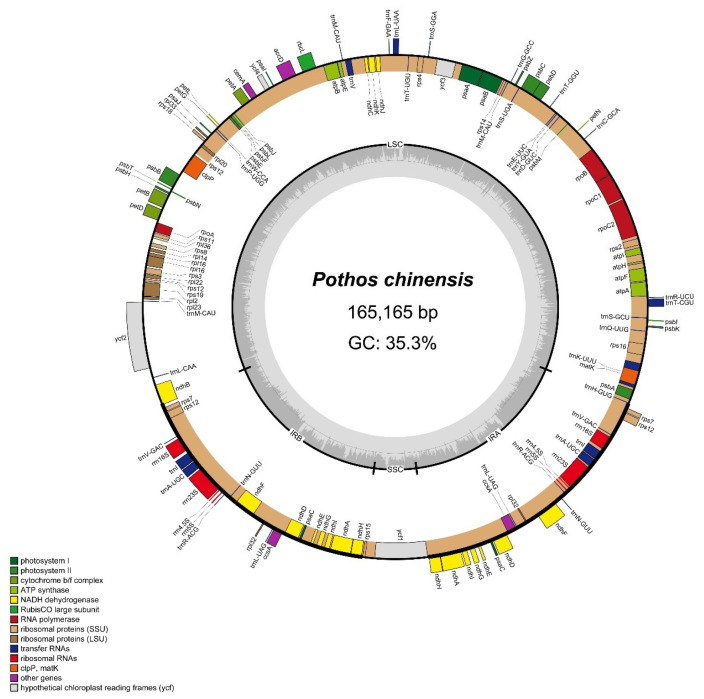
Chloroplast genome map of *P. chinensis*. Genes positioned in the outer circle are transcribed in a counterclockwise manner, whereas genes in the inner circle are transcribed in the clockwise direction. Within the inner circle, dark gray regions correspond to segments with elevated GC content, while lighter gray areas indicate higher AT content.

The sequencing of 150 bp single-end reads generated 7.99 GB data (15.15 million reads). The chloroplast reads were used for de novo assembly and provided average coverage depths of 178.2× for *P. chinensis*. The length of the complete chloroplast genome of *P. chinensis* (PV938952) is 165,165 bp and it is a typical quadripartite structure, with a small single-copy region (SSC) of 6847 bp, a large single-copy region (LSC) of 103,234 bp, and a pair of IRs of 27,542 bp (Figure 1). There are 134 genes annotated, including 90 protein-coding genes, 36 transfer RNA (tRNAs) genes, and eight ribosomal RNA (rRNAs) genes (Table S2). Within the 134 genes, 72 were related to self-replication, including 10 genes related to the large subunit of the ribosome and 14 related to the small subunit of the ribosome. A total of 52 genes were involved in photosynthesis, including 6 related to ATP synthase, 18 to NADH dehydrogenase, 6 to the cytochrome b/f complex, 6 to the PS I system, 15 to the PS II system, and 1 associated with Rubisco. Additionally, 10 genes were annotated as having other functions (*clpP*, *ccsA*, *accD*, *cemA*, and *matK*) or unknown functions (*ycf1*, *ycf2*, *ycf3*, and *ycf4*). In total, 13 genes had a single intron (*atpF*, *ndhA*, *ndhB*, *petB*, *petD*, *rpl2*, *rpoC1*, *rps12*, *trnA^UGC^*, *trnI^GAU^*, *trnK^UUU^*, *trnL^UAA^,* and *trnV^UAC^*), while 2 genes (*clpP* and *ycf3*) contained two introns (Table S2).

### 3.2. Structural Variations, Sequence Divergence, and Nucleotide Diversity

**Figure 2:**
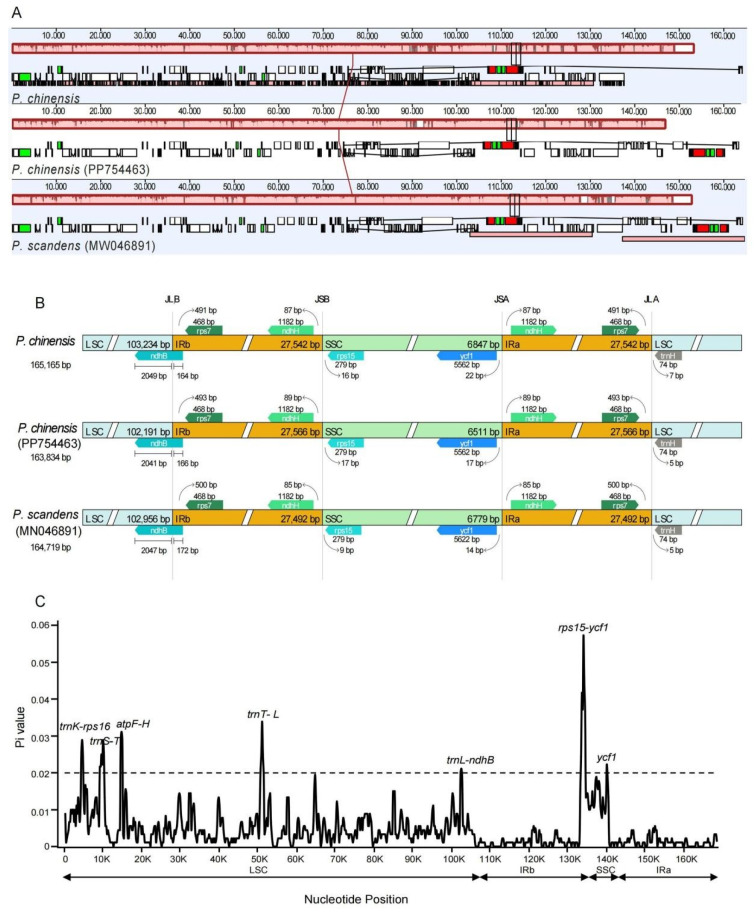
Analysis of collinearity, boundary characteristics, and nucleotide diversity of chloroplast genomes within *Pothos*. (**A**) Collinearity analysis of chloroplast genomes. The same color represents homologous segments between different chloroplast genomes and were connected by lines. (**B**) Four junctions in the chloroplast genomes of different *Pothos* species. (**C**) Nucleotide diversity analysis. The horizontal axis shows the nucleotide positions, and the vertical axis displays the corresponding Pi values.

The collinearity analysis revealed no gene rearrangements or inversions in the three *Pothos* chloroplast genomes (Figure 2A). The IR boundary map showed that the *ndhB* gene in three *Pothos* individuals spanned from LSC to IRb with 164–172 bp departed in IRb (Figure 2B). Moreover, the *ndhH* and *rps15* genes were found adjacent to the junction between the SSC and IRb (JSB), while none of them spanned the junction. Also, *ycf1* and *ndhH* were found adjacent to the junction between the LSC and IRa (JSA) (Figure 2B). To further explore the mutational hotspots to develop the specific DNA barcodes of *Pothos* species, the results of nucleotide diversity (Pi) values are shown in Figure 2C. According to the ranking of Pi values, the seven hypervariable regions of whole chloroplast genomes were identified: *trnK-rps16*, *trnS-T*, *atpF-H*, *trnT-L*, *trnL-ndhB*, *rps15-ycf1*, and *ycf1*.

### 3.3. Phylogenetic Analysis

**Figure 3:**
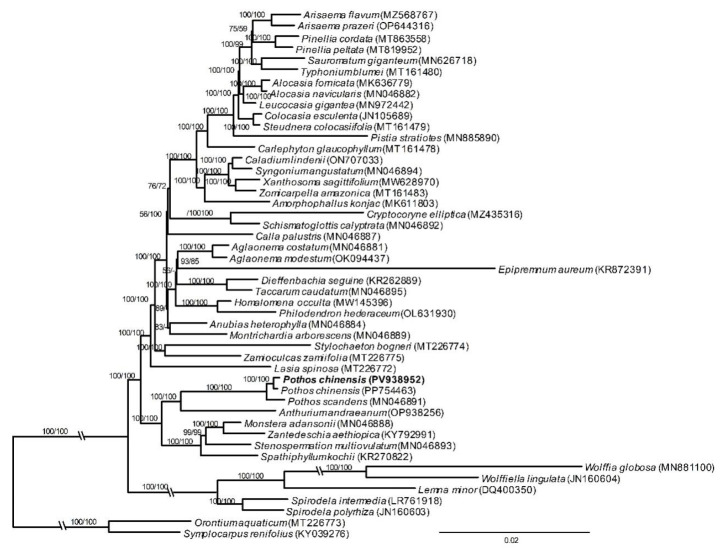
Phylogenetic tree of Araceae based on chloroplast genomes. The numbers on branches represented the bootstrap values of ML and MP, respectively.

Both ML and MP analyses showed that *P. chinensis* was a sister group of *A. andraeanum* (ML-BP = 100%, BI-PP = 1.00), and the sampled *Pothos* species formed a clade with the highest supporting values (ML-BP = 100%, BI-PP = 1.00). Among them, *P. chinensis* was closely related to *P. scandens* with strongly supported values (ML-BP = 100%, BI-PP = 1.00, Figure 3).

### 3.4. Codon Usage Bias and the Optimal Codons

The ENC values in the chloroplast genome of *P. chinensis* ranged from 34.72 (for *rps18*) to 54.41 (for *clpP*), with an average of 45.49. Among the 50 genes, only the ENC value of *rps18* was less than 35, which indicated a relatively weak codon usage bias in the chloroplast genome of *P. chinensis* (Table S3). The overall GC content across all genes (GC_all) of *P. chinensis* ranged from 30.20% (for *ycf1*) to 44.44% (for *psbC*), with an average of 38.34% (Table S3). The GC content at the first, second, and third codon positions (GC1, GC2, and GC3) ranged from 32.92% (for *ccsA*) to 57.79% (for *rbcL*), 28.53% (for *ycf1*) to 55.40% (for *rps11*), and 20.59% (for *rps18*) to 51.94% (for *ycf2*), respectively (Table S3). The averages of GC1, GC2, and GC3 were 47.02%, 39.80%, and 28.20%, respectively (Table S3). GC_all showed a highly significant positive correlation with GC1, GC2, and GC3 at the 0.01 level. GC1 and GC2 were significantly positively correlated, but GC3 did not show a significant correlation with either GC1 or GC2. ENC had a highly significant positive correlation with GC3 and a significant positive correlation with GC1, yet no relevant correlation with GC2. This indicated that the codon usage bias in the chloroplast genome of *P. chinensis* was primarily influenced by GC3 and GC1, with relatively minor influences from GC2 (Table 1).

**Figure 4:**
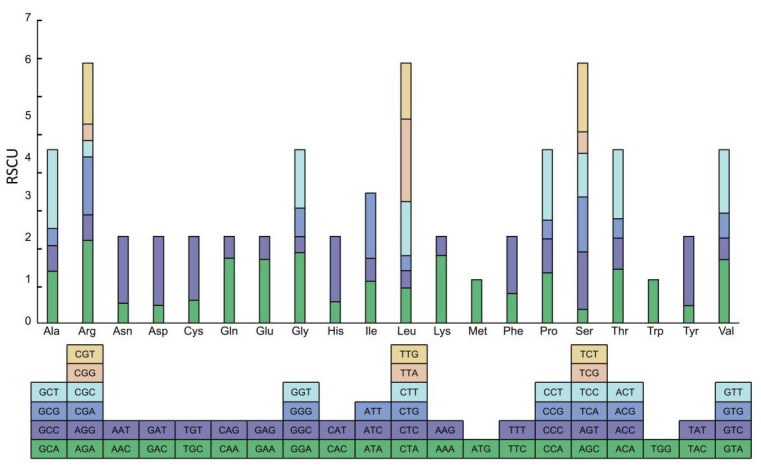
Analysis of relative synonymous codon usage of chloroplast genes in *P. chinensis*. Except for the RSCU values of methionine and tryptophan that are equal to 1, the RSCU values of the 32 codons are greater than 1, and those of 30 codons are less than 1.

There were 14 high-expression preferred codons with ΔRSCU ≥ 0.08 (Table 2), and 32 high-frequency codons with RSCU > 1 (Figure 4). The codons that simultaneously meet the conditions of being both high-expression preferred and high-frequency were considered the optimal codons; therefore, 14 codons were selected as the optimal codons (including GCU, AGA, CAA, AUU, UUA, UUG, AAA, CCU, AGU, UCC, UCG, ACU, GUA, and GUU). Among these codons, the GCU codon had the highest ΔRSCU value (0.80; Table 2). There were 11 codons ending with A/U and 3 codons ending with G/C, indicating a preference for optimal codons in *P. chinensis* to end with A/U bases.

### 3.5. Neutrality Plot Analysis

**Figure 5:**
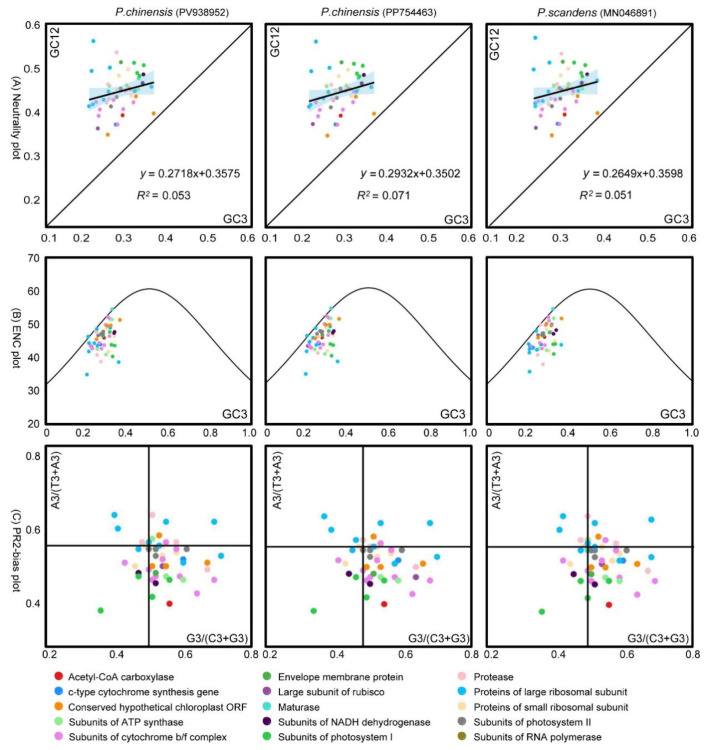
Analysis of the causes of codon preference in *P. chinensis*. (**A**) Neutrality plot analysis. The black line represents the correlation trend, with the corresponding values at the bottom of the plot. (**B**) ENC-plot analysis. When a data point is far-distant from the standard curve, it indicates that the codon usage bias is predominantly influenced by natural selection. (**C**) PR2-plot analysis. The GC bias is plotted on the horizontal -axis, while the AT bias is plotted on the vertical -axis.

The neutrality plot of the chloroplast genome of *P. chinensis* (PV938952) revealed that all of the genes were distributed above the diagonal (Figure 5A). The GC3 distribution ranged from 20.59 to 35.64, while the GC12 distribution ranged from 32.74 to 55.76. Correlation analysis between GC3 and GC12 yielded a *p*-value of 0.2300, with a regression coefficient of 0.2718, which represented a weak relationship between them. The contribution rate of internal mutation was 27.18%, while that of natural selection was 72.82%. Moreover, the regression coefficient of *P. chinensis* (PP754463) was 0.2932, and that of *P. scandens* was 0.2649. These results indicate that natural selection might have a stronger impact on codon preference in the chloroplast genomes of the genus *Pothos* than internal mutational pressure.

### 3.6. ENC-Plot Analysis

**Figure 6:**
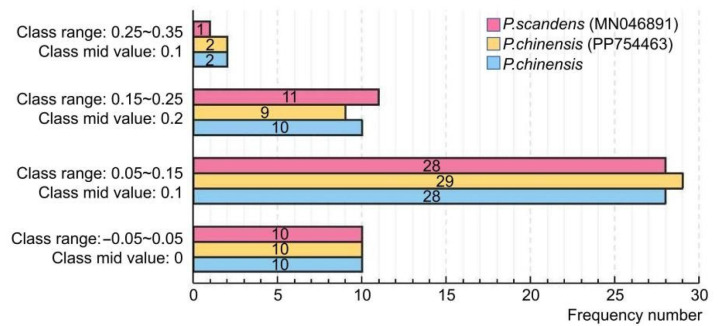
Distribution of ENC ratio frequency.

The results of the ENC-plot analysis of the three *Pothos* chloroplast genomes are shown in Figure 5B. The majority of the genes in the three plant chloroplast genomes were located below the standard curve, suggesting that the ENC_obs_ values of these genes differ substantially from the ENC_exp_. A subsequent statistical analysis of the ENC ratios revealed that only 10 genes in each genome fall within the range of −0.05 to 0.05, while 40 genes exhibited ENC ratios outside this range (Figure 6). Collectively, these findings indicated that the codon preference in the three *Pothos* chloroplast genomes was more influenced by natural selection than internal mutational pressure.

### 3.7. Parity-Rule 2 (PR2) Bias Plot Analysis

Codon preference in the chloroplast genome was conducted through PR2-plot graphing (Figure 5C). The coding genes were unevenly distributed across the four regions of the plot, with some points farther away from the axes in all the three chloroplast genomes. There was a higher density of genes in the lower right quadrant of the plot. Specifically, there were 37 points in *P. chinensis* (PV938952) with G3/(G3 + C3) > 0.5 and 36 points with A3/(A3 + T3) < 0.5. In *P. chinensis* (PP754463)*,* these corresponding numbers were 35 and 36, respectively. In *P.scandens* (MN046891), there were 34 points with G3/(G3 + C3) > 0.5 and 36 points with A3/(A3 + T3) < 0.5. These results indicated that in the three chloroplast genomes, the frequency of the third base usage in codons is U > A and G > C. This also suggests that the codon usage pattern in *P. chinensis* is subject to natural selection.

## 4. Discussion

### 4.1. Chloroplast Genome Evolution Within P. chinensis

In this study, we screened the chloroplast genome of *P. chinensis* and compared the chloroplast characters at the interspecific and intraspecific level, which provided genetic resources for understanding the evolution of chloroplast genomes in this group. The chloroplast genome of *P. chinensis* has a typical quadripartite structure, consisting of one LSC, one SSC, and two IR regions, which were similar to the other Araceae species and the angiosperm [10]. The lengths of LSC, SSC, and two IRs are similar within *P. chinensis* and *P. scandens* (Figure 2), which indicated that the chloroplast genome characters may be conserved within *Pothos*. Further, the length of the *P. chinensis* individual obtained in this study (PV938952, 165,165 bp) is slightly bigger than the other one (PP754463, 163,834 bp, Figure 2B), and this reflects the genetic diversity of the chloroplast genome within the different individuals of the same species. However, compared to its sister group *A. andraeanum*, the SSC length of *P. chinensis* was obviously shorter, while the LSC length was bigger. The results were similar with those among *P. scandens* and *A. andraeanum* [11].

No visible gene rearrangement was detected, and all IR boundaries were conserved without obvious expansion or contraction within the two *Pothos* species (Figure 2B). The genes at the boundary of JLB, JSB, JSA, and JLA show consistency, only with a slight length difference. In addition, our results detected seven hypervariable regions of whole plastomes (*trnK-rps16*, *trnS-T*, *atpF-H*, *trnT-L*, *trnL-ndhB*, *rps15-ycf1*, and *ycf1*) within *Pothos* (Figure 2C), most of which were also proposed in previous studies to resolve the phylogenetic relationships among the related species [43,44,45]. Therefore, we consider that the seven hypervariable regions of the whole chloroplast genome might be powerful DNA markers for the phylogenetic analysis of *Pothos*.

### 4.2. Natural Selection and the Codon Preference of P. chinensis Chloroplast Genome

Synonymous codons exhibit a certain degree of preference in their usage across different species and among different genes within the same species. Genes with high expression levels typically possess optimal codons and demonstrate stronger codon usage bias [46,47,48,49,50]. In this study, GC3 in the chloroplast genome of *P. chinensis* exhibited no significant correlation with GC1 or GC2, and the proportion of GC3 was the lowest among the three (Table 1). Furthermore, the codon usage bias in the chloroplast genome of *P. chinensis* was found to predominantly end with A/U nucleotides (Table 2, Figure 5C). A similar pattern was detected in other monocots, such as the Poaceae and *Lilium* species [51,52]. The degree of codon preference can be determined using the ENC value. When the ENC is greater than 35, it indicates that the codon preference is relatively weak and vice versa [36]. This study found that most ENC values in the chloroplast genome of *P. chinensis* are greater than 35, indicating a relatively weak codon usage preference in the chloroplast genome of *P. chinensis*.

To gain a clearer understanding of the factors influencing codon usage preference, this study conducted neutral plot, ENC-plot, and PR2-plot analyses on the chloroplast genome codons of *P. chinensis* (Figure 5). In the neutral plot analysis, the regression coefficient for *P. chinensis* was 0.2718, indicating a stronger influence by natural selection than mutation. In the ENC-plot analysis, most genes in *P. chinensis* were significantly distant from the standard curve, with actual ENC values differing from expected ENC values, suggesting that the codon preference characteristics of these genes are primarily constrained by natural selection. In the PR2-plot analysis, most genes in the chloroplast genome of *P. chinensis* were located in the lower right quadrant of the plot, indicating different usage frequencies among the four bases, specifically T > A and G > C. This also suggests that codon usage is more influenced by natural selection. These analytic data lead us to conclude that the main factor influencing codon usage preference in the chloroplast genome of *P. chinensis* is natural selection. This result is consistent with the studies on the codon usage preference of chloroplast genomes in some monocotyledonous plants [50,51] and dicotyledonous plants [52,53]. Furthermore, 14 optimal codons were screened using RSCU and ENC values (Figure 4 and Table 2), and most of the optimal codons ended with A or U. In future research, the reasons behind the influence of natural selection upon codon usage bias should be explored.

## 5. Conclusions

In this study, the whole chloroplast genome of *P. chinensis* was newly obtained, and its GC content and CDS content characteristics are conserved. Compared to the sister group *A. andraeanum*, the length of LSC and SSC in *P. chinensis* and *P. scandens* expanded and contracted, respectively. According to Pi values, seven hypervariable regions (*trnK*-*rps16*, *trnS-T*, *atpF-H*, *trnT-L*, *trnL-ndhB*, *rps15-ycf1*, and *ycf1*) were identified. Both GC content and ENC values revealed that the codon usage bias of the *P. chinensis* chloroplast genome was weak, and the codon usage bias was found to predominantly end with A/U. Further, the results of neutral plot, PR2-plot, and ENC-plot concluded that natural selection is the main factor influencing codon usage bias in the chloroplast genome of *P. chinensis*. Fourteen optimal codons were identified in the chloroplast genome of *P. chinensis*. These findings might play an essential role in the chloroplast evolutionary dynamics of *Pothos*.

## Figures and Tables

**Table 1:** Correlation analysis of codon parameters in *P. chinensis* chloroplast genes.

Variation	GC1	GC2	GC3	GC_all	ENC
GC2	0.361 *				
GC3	0.247	0.125			
GC_all	0.813 **	0.727 **	0.558 **		
ENC	0.303 *	−0.200	0.387 **	0.210	
Codon No.	−0.153	−0.310 *	0.216	−0.157	0.256

** *p* < 0.01; * *p* < 0.05.

**Table 2:** Optimal codons in the chloroplast genome of *P. chinensis*.

Amino Acid	Codon	High Expression Gene		Low Expression Gene		∆RSCU
		RSCU	No.	RSCU	No.	
Ala	GCU ***	2.45	38	1.65	42	0.80
Arg	AGA *	2.16	31	1.97	65	0.19
Gln	CAA *	1.46	19	1.34	90	0.12
Ile	AUU *	1.48	34	1.30	110	0.18
Leu	UUA *	1.46	17	1.19	64	0.27
	UUG *	1.63	19	1.53	82	0.10
Lys	AAA **	1.65	28	1.22	118	0.43
Pro	CCU ***	2.11	19	1.44	57	0.67
Ser	AGU ***	1.77	18	1.00	52	0.77
	UCC *	1.28	13	1.18	61	0.10
	UCG *	1.77	18	1.56	81	0.21
Thr	ACU ***	1.95	21	1.44	50	0.51
Val	GUA ***	1.62	15	1.04	40	0.58
	GUU **	1.95	18	1.48	57	0.47

* 0.08 ≤ ∆ RSCU < 0.30, ** 0.30 ≤ ∆ RSCU < 0.50, *** 0.50 ≤ ∆ RSCU.

## Supplementary Materials

The following supporting information can be downloaded at: https://www.mdpi.com/article/10.3390/genes16091017/s1, Table S1: Taxa, voucher and GenBank accession numbers used in this study; Table S2: Group of chloroplast genome genes in *P*. *chinensis*; Table S3: Basic parameters of codon usage bias of *P. chinensis* chloroplast genes.
