# Supplementary material for: Chloroplast Genome Evolution and Codon Usage In the Medicinal Plant Pothos chinensis (Araceae)
Source: Genes (Basel). 2025 Aug 28;16(9):1017. doi: 10.3390/genes16091017 (PMC12470003; doi:10.3390/genes16091017)
Supplement: Supplementary file 1 [file genes-16-01017-s001.zip › genes-3818234-supplementary.pdf]

**Supplementary materials**

**Table S1.** Taxa, voucher and GenBank accession numbers used in this study.

**Table S2.** Group of chloroplast genome genes in *P. chinensis.*

**Table S3.** Basic parameters of codon usage bias of *P. chinensis* chloroplast genes.

**Table S1** Taxa, voucher and GenBank accession numbers used in this study.

| Taxon | GenBank Accession No. | Voucher |
|---|---|---|
| *Acorus calamus* | MT755635 | |
| *Aglaonema costatum* | MN046881 | |
| *Aglaonema modestum* | OK094437 | |
| *Alocasia fornicata* | MK636779 | |
| *Alocasia navicularis* | MN046882 | |
| *Amorphophallus konjac* | MK611803 | |
| *Anthurium andraeanum* | OP938256 | |
| *Anubias heterophylla* | MN046884 | |
| *Arisaema flavum* | MZ568767 | |
| *Arisaema prazeri* | OP644316 | |
| *Caladium lindenii* | ON707033 | |
| *Calla palustris* | MN046887 | |
| *Carlephyton glaucophyllum* | MT161478 | |
| *Colocasia esculenta* | JN105689 | |
| *Cryptocoryne elliptica* | MZ435316 | |
| *Dieffenbachia seguine* | KR262889 | |
| *Epipremnum aureum* | KR872391 | |
| *Homalomena occulta* | MW145396 | |
| *Lasia spinosa* | MT226772 | |
| *Lemna minor* | DQ400350 | |
| *Leucocasia gigantea* | MN972442 | |
| *Monstera adansonii* | MN046888 | |
| *Montrichardia arborescens* | MN046889 | |
| *Orontium aquaticum* | MT226773 | |
| *Philodendron hederaceum* | OL631930 | |
| *Pinellia cordata* | MT863558 | |
| *Pinellia peltata* | MT819952 | |
| *Pistia stratiotes* | MN885890 | |
| *Pothos chinensis* | PV938952 | Zhang JS, ZJS_2023070 (Guizhou) |
| *Pothos chinensis* | PP754463 | |
| *Pothos scandens* | MN046891 | |
| *Sauromatum giganteum* | MN626718 | |
| *Schismatoglottis calyptrata* | MN046892 | |
| *Spathiphyllum kochii* | KR270822 | |
| *Spirodela intermedia* | LR761918 | |
| *Spirodela polyrhiza* | JN160603 | |
| *Stenospermation multiovulatum* | MN046893 | |
| *Steudnera colocasiifolia* | MT161479 | |
| *Stylochaeton bogneri* | MT226774 | |
| *Symplocarpus renifolius* | KY039276 | |
| *Syngonium angustatum* | MN046894 | |
| *Taccarum caudatum* | MN046895 | |

| | |
|---|---|
| *Thepparatia scandens* | OQ471924 |
| *Typhonium blumei* | MT161480 |
| *Wolffia globosa* | MN881100 |
| *Wolffiella lingulata* | JN160604 |
| *Xanthosoma sagittifolium* | MW628970 |
| *Zamioculcas zamiifolia* | MT226775 |
| *Zantedeschia aethiopica* | KY792991 |
| *Zomicarpella amazonica* | MT161483 |

**Table S2** Group of chloroplast genome genes in *P. chinensis*.

| Category | Group of Genes | Name of Genes |
|---|---|---|
| Self-replication | Ribosomal RNA | *rrn4.5**, *rrn5**, *rrn16**, *rrn23** |
| | Transfer RNA | *trnA-UGC**,†, *trnC-GCA*, *trnD-GUC*, *trnE-UUC*, *trnF-GAA*, *trnM-CAU**, *trnG-GCC*, *trnH-GUG*, *trnI-GAU**,†, *trnK-UUU*†, *trnL-CAA*, *trnL-UAA*†, *trnL-UAG**, *trnN-GUU**, *trnP-UGG*, *trnQ-UUG*, *trnR-ACG**, *trnR-UCU*, *trnS-GCU*, *trnS-GGA*, *trnS-UGA*, *trnT-CGU*, *trnT-GGU*, *trnT-UGU*, *trnV-GAC**, *trnV-UAC*†, *trnW-CCA*, *trnY-GUA* |
| | Small subunit of ribosome | *rps2*, *rps3*, *rps4*, *rps7**, *rps8*, *rps11*, *rps12**,†, *rps14*, *rps15*, *rps16*, *rps18*, *rps19* |
| | Large subunit of ribosome | *rpl2*†, *rpl14*, *rpl16*, *rpl20*, *rpl22*, *rpl23*, *rpl32**, *rpl33*, *rpl36* |
| | RNA polymerase subunit | *rpoA*, *rpoB*, *rpoC1*†, *rpoC2* |
| Photosynthesis | ATP synthase | *atpA*, *atpB*, *atpE*, *atpF*†, *atpH*, *atpI* |
| | NADH dehydrogenase | *ndhA**,†, *ndhB*†, *ndhC*, *ndhD**, *ndhE**, *ndhF**, *ndhG**, *ndhH**, *ndhI**, *ndhJ*, *ndhK* |
| | Cytochrome b/f complex | *petA*, *petB*†, *petD*†, *petG*, *petL*, *petN* |
| | Photosystem I | *psaA*, *psaB*, *psaC**, *psaI*, *psaJ* |
| | Photosystem II | *psbA*, *psbB*, *psbC*, *psbD*, *psbE*, *psbF*, *psbH*, *psbI*, *psbJ*, *psbK*, *psbL*, *psbM*, *psbN*, *psbT*, *psbZ* |
| | Rubisco large subunit | *rbcL* |
| Others | Proteolysis | *clpP*† |
| | Cytochrome *c* biogenesis protein | *ccsA** |
| | Acetyl-CoA carboxylase | *accD* |
| | Chloroplast envelope membrane protein | *cemA* |
| | maturase | *matK* |
| | Hypothetical reading frame | *ycf1*, *ycf2*, *ycf3*†, *ycf4* |

*, duplicated gene in IR; †, gene including one or two introns

**Table S3** Basic parameters of codon usage bias of *P. chinensis* chloroplast genes.

| Genes | codon No. | GC1 | GC2 | GC3 | GC_all | ENC | $GC3_s$ |
|---|---|---|---|---|---|---|---|
| *accD* | 495 | 38.18 | 36.97 | 28.69 | 34.61 | 45.79 | 28.8 |
| *atpA* | 508 | 54.92 | 39.57 | 27.76 | 40.75 | 47.47 | 28.0 |
| *atpB* | 499 | 56.51 | 41.08 | 30.06 | 42.55 | 48.60 | 30.4 |
| *atpE* | 137 | 51.09 | 40.15 | 33.58 | 41.61 | 46.29 | 33.6 |
| *atpF* | 182 | 48.90 | 35.71 | 30.77 | 38.46 | 41.64 | 29.9 |
| *atpI* | 248 | 50.40 | 37.10 | 27.42 | 38.31 | 46.35 | 27.4 |
| *ccsA* | 322 | 32.92 | 37.58 | 27.02 | 32.51 | 43.79 | 27.0 |
| *clpP* | 203 | 56.65 | 36.45 | 32.51 | 41.87 | 54.41 | 32.0 |
| *matK* | 516 | 38.18 | 30.43 | 22.67 | 30.43 | 43.16 | 22.9 |
| *ndhA* | 364 | 43.41 | 38.19 | 22.25 | 34.62 | 43.37 | 22.7 |
| *ndhB* | 511 | 42.47 | 38.94 | 31.31 | 37.57 | 46.31 | 31.5 |
| *ndhC* | 121 | 47.93 | 34.71 | 26.45 | 36.36 | 44.24 | 25.8 |
| *ndhE* | 102 | 38.24 | 32.35 | 27.45 | 32.68 | 42.54 | 27.3 |
| *ndhF* | 742 | 37.87 | 36.93 | 22.91 | 32.57 | 42.64 | 23.2 |
| *ndhG* | 177 | 44.63 | 33.33 | 28.81 | 35.59 | 43.58 | 29.0 |
| *ndhH* | 394 | 51.52 | 35.79 | 29.44 | 38.92 | 49.67 | 30.0 |
| *ndhI* | 181 | 41.44 | 36.46 | 23.20 | 33.70 | 42.57 | 22.0 |
| *ndhJ* | 159 | 49.06 | 37.74 | 31.45 | 39.41 | 51.52 | 31.8 |
| *ndhK* | 226 | 42.48 | 45.58 | 30.53 | 39.53 | 52.03 | 31.1 |
| *petA* | 321 | 52.65 | 35.20 | 32.40 | 40.08 | 51.32 | 31.7 |
| *petB* | 216 | 48.15 | 41.67 | 29.63 | 39.81 | 40.74 | 29.8 |
| *petD* | 161 | 50.31 | 39.13 | 25.47 | 38.30 | 42.38 | 25.8 |
| *psaA* | 751 | 51.26 | 43.81 | 33.69 | 42.92 | 47.55 | 33.8 |
| *psaB* | 735 | 47.89 | 42.86 | 33.47 | 41.41 | 47.21 | 33.8 |
| *psbA* | 354 | 50.28 | 44.07 | 32.77 | 42.37 | 40.16 | 32.9 |
| *psbB* | 509 | 54.42 | 46.37 | 31.43 | 44.07 | 47.53 | 31.4 |
| *psbC* | 474 | 53.38 | 46.20 | 33.76 | 44.44 | 43.44 | 33.6 |
| *psbD* | 354 | 52.26 | 43.50 | 32.20 | 42.66 | 43.67 | 32.2 |
| *rbcL* | 488 | 57.79 | 43.24 | 28.28 | 43.10 | 46.27 | 28.3 |
| *rpl14* | 123 | 53.66 | 36.59 | 25.20 | 38.48 | 47.51 | 25.4 |
| *rpl16* | 136 | 51.47 | 54.41 | 27.21 | 44.36 | 38.59 | 28.8 |
| *rpl20* | 118 | 37.29 | 45.76 | 25.42 | 36.16 | 40.61 | 26.4 |
| *rpl22* | 133 | 42.86 | 38.35 | 24.81 | 35.34 | 47.93 | 24.2 |
| *rpoA* | 340 | 45.59 | 33.82 | 25.88 | 35.10 | 46.45 | 26.0 |
| *rpoB* | 1073 | 49.67 | 37.56 | 28.33 | 38.52 | 47.68 | 28.6 |
| *rpoC1* | 684 | 50.29 | 37.87 | 28.22 | 38.79 | 46.95 | 28.4 |
| *rpoC2* | 1354 | 46.53 | 37.44 | 27.25 | 37.08 | 46.93 | 27.2 |
| *rps11* | 139 | 56.12 | 55.40 | 21.58 | 44.36 | 44.19 | 21.2 |
| *rps12* | 133 | 51.13 | 47.37 | 25.56 | 41.35 | 43.41 | 26.0 |
| *rps14* | 101 | 42.57 | 46.53 | 35.64 | 41.58 | 38.46 | 35.5 |
| *rps18* | 102 | 38.24 | 41.18 | 20.59 | 33.33 | 34.72 | 20.2 |
| *rps2* | 237 | 43.04 | 43.88 | 31.65 | 39.52 | 48.83 | 31.7 |

| | | | | | | | |
|---|---|---|---|---|---|---|---|
| *rps3* | 219 | 47.49 | 32.88 | 21.46 | 33.94 | 41.67 | 22.0 |
| *rps4* | 202 | 48.02 | 38.61 | 25.25 | 37.29 | 48.45 | 25.6 |
| *rps7* | 156 | 51.92 | 44.87 | 21.15 | 39.32 | 46.15 | 21.6 |
| *rps8* | 133 | 40.60 | 42.11 | 24.06 | 35.59 | 44.17 | 22.8 |
| *ycf1* | 1854 | 36.95 | 28.53 | 25.13 | 30.20 | 45.34 | 25.2 |
| *ycf2* | 2303 | 41.42 | 34.61 | 36.21 | 37.41 | 51.18 | 36.4 |
| *ycf3* | 169 | 47.93 | 40.24 | 30.18 | 39.45 | 49.43 | 30.7 |
| *ycf4* | 185 | 43.24 | 41.08 | 31.89 | 38.74 | 49.51 | 31.5 |
| Average | 407 | 47.02 | 39.80 | 28.20 | 38.34 | 45.49 | 28.26 |
